# Hemophagocytic Lymphohistiocytosis as the Presenting Manifestation of Relapsed Classic Hodgkin’s Lymphoma in the Presence of Concurrent Human Immunodeficiency Virus, Genital Herpes, Epstein-Barr Virus and Mycobacterium Avium Complex Infection

**DOI:** 10.7759/cureus.11563

**Published:** 2020-11-19

**Authors:** Raheel S Siddiqui, Mariam Agladze, Tayyaba Bashir

**Affiliations:** 1 Internal Medicine, Icahn School of Medicine at Mount Sinai (New York City Health and Hospitals/Queens), Jamaica, USA; 2 Internal Medicine, Icahn School of Medicine at Mount Sinai, Queens Hospital Center, Jamaica, USA; 3 Hematology/Oncology, Icahn School of Medicine at Mount Sinai (New York City Health and Hospitals/Queens), Jamaica, USA

**Keywords:** hemophagocytic lymphohistiocytosis (hlh), secondary hemophagocytic lymphohistiocytosis, classic hodgkin lymphoma, relapse lymphoma

## Abstract

Hemophagocytic lymphohistiocytosis (HLH) is a life-threatening disorder of uncontrolled immune activation which is usually divided into two main types. Primary, which is associated with genetic mutation and familial predisposition and secondary, which is usually associated with infections, malignancies and autoimmune conditions. More often multiple risk factors are present at the time of initial presentation. We report a case where HLH was the presenting manifestation of relapsed Classic Hodgkin’s Lymphoma in the presence of multiple risk factors of secondary HLH such as human immunodeficiency virus (HIV), active genital herpes, Epstein-Barr virus (EBV) viremia, Mycobacterium avium complex (MAC) infection and prior chemotherapy. A 38-year-old male to female transgender woman presented with one-week history of fever, nausea, vomiting and generalized weakness. The past medical history was significant for HIV and previously treated and positron emission tomography (PET) scan confirmed complete remission of Classic Hodgkin’s Lymphoma. Physical examination showed BP 92/40 mmHg, fever of 102.6 F, heart rate of 114 beats per minutes, diffuse abdominal tenderness and male genitalia with multiple small ulcerative lesions. Labs showed pancytopenia, hyponatremia, mildly elevated total and direct bilirubin, transaminitis, CD-4 count 96/mcL, HIV viral load undetectable and COVID-19 polymerase chain reaction (PCR) negative. Imaging showed right middle lung lobe consolidation and hepatosplenomegaly with multiple hypodense lesions. Lymphadenopathy was reported in mediastinum and retroperitoneum. The patient was initially treated with broad spectrum antibiotics, IV fluids, vasopressors and stress dose steroids. After initial improvement, vasopressors and steroids were stopped. The patient again started spiking fever on day 9 despite being on antibiotics. Workup showed EBV viremia, genital herpes and evidence of MAC infection on sputum culture. No improvement noted despite appropriate treatment for genital herpes and MAC. Additional lab work showed hyperferritinemia and elevated soluble Interleukin-2 receptors. The patient was diagnosed with HLH as per HLH-2004 criteria and treated with dexamethasone and etoposide. Bone marrow biopsy confirmed hemophagocytosis and immunoperoxidase staining established the diagnosis of relapsed Classic Hodgkin’s Lymphoma. We can conclude that in patients with a history of hematological malignancy presenting with HLH, a high degree of suspicion for relapse should be maintained even in the presence of other risk factors.

## Introduction

Hemophagocytic lymphohistiocytosis (HLH) is a life-threatening disorder of uncontrolled immune activation. It is divided into two main types. Primary, which is usually a disease of childhood and associated with genetic mutations and familial predisposition. Secondary, which is more common in adults and associated with infections, malignancies, and autoimmune conditions [1].

HLH usually presents with fever, cytopenia (bi- or pancytopenia) and hepatosplenomegaly. Other common laboratory findings include hyperferritinemia, hypertriglyceridemia, elevated liver enzymes, hypofibrinogenemia, coagulopathy. Less common findings include lymphadenopathy, neurological changes, skin rash, jaundice and edema [1].

The initial presentation of HLH often mimics sepsis and septic shock sometimes necessitating intensive care unit (ICU) admission and vasopressor support [2]. The persistence of symptoms without any definitive source of infection should raise the suspicion of HLH. It is important to keep high suspicion of index due to high morbidity and mortality associated with this condition and earlier diagnosis and treatment improves the outcomes.

Secondary HLH is diagnosed by clinical criteria used in HLH-2004 study [1] which requires presence of five or more of the eight clinical findings which include: fever, cytopenia (bicytopenia or pancytopenia), splenomegaly, hypertriglyceridemia and/or hypofibrinogenemia, evidence of hemophagocytosis in bone marrow, lymph node or spleen, hyperferritinemia, elevated soluble CD25 (Interleukin-2 Receptor) and low or absent natural killer cell activity. The last three criteria were absent in 1991 guidelines and were added in a 2004 study.

The most common inciting factor for secondary HLH in adults is infection followed by malignancy and autoimmune conditions. Many patients with secondary HLH have multiple risk factors [2-6]. It is important to diagnose not only HLH but also the inciting event as the treatment considerations and prognosis greatly depends on the underlying cause [7].

We report a case where HLH was the presenting manifestation of relapsed Classic Hodgkin’s Lymphoma in the presence of multiple risk factors of secondary HLH such as human immunodeficiency virus (HIV) infection, active genital herpes/herpes simplex virus-2 (HSV-2) infection, Epstein-Barr virus (EBV) viremia, Mycobacterium avium complex (MAC) infection and prior chemotherapy. Very limited data is available on patients with Hodgkin’s Lymphoma who presented with HLH. To the best of our knowledge, this is the first case where HLH is reported with relapsed Classic Hodgkin's Lymphoma in the presence of HIV, EBV, HSV-2, MAC and prior chemotherapy.

## Case presentation

**Figure 1:**
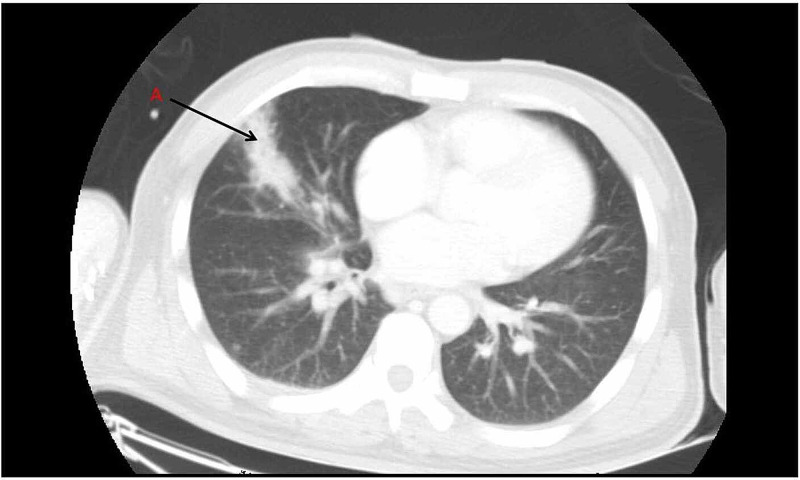
CT chest showing right lung opacity A: Right middle lobe opacity

**Figure 2:**
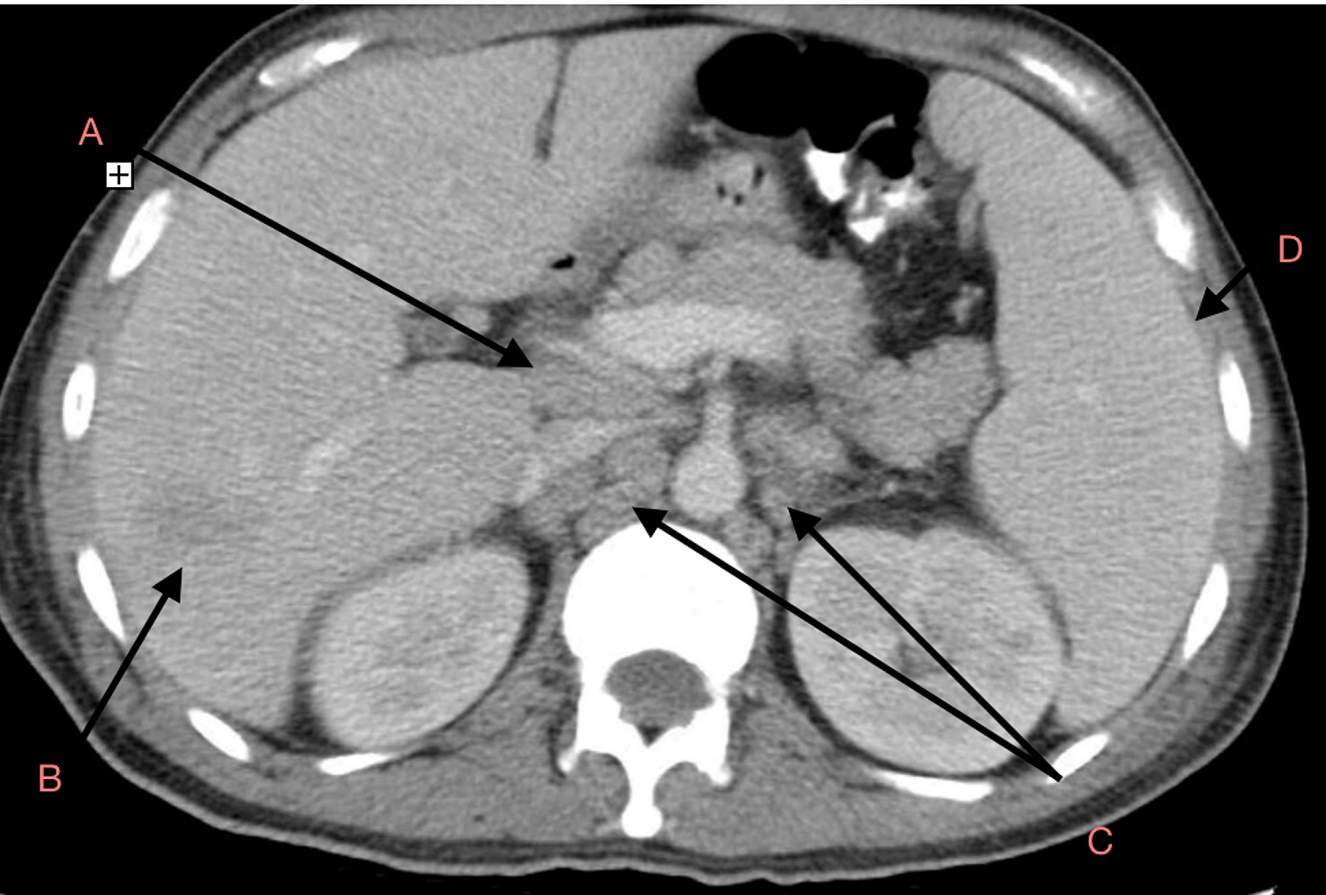
CT abdomen showing hypodense lesion in the liver, splenomegaly and retroperitoneal lymphadenopathy A: Portocaval Lymph Nodes, B: Right Posterior Hepatic Lobe Hypodense Lesion, C: Paraaortic Retroperitoneal Lymphadenopathy, D: Enlarged Spleen

**Figure 3:**
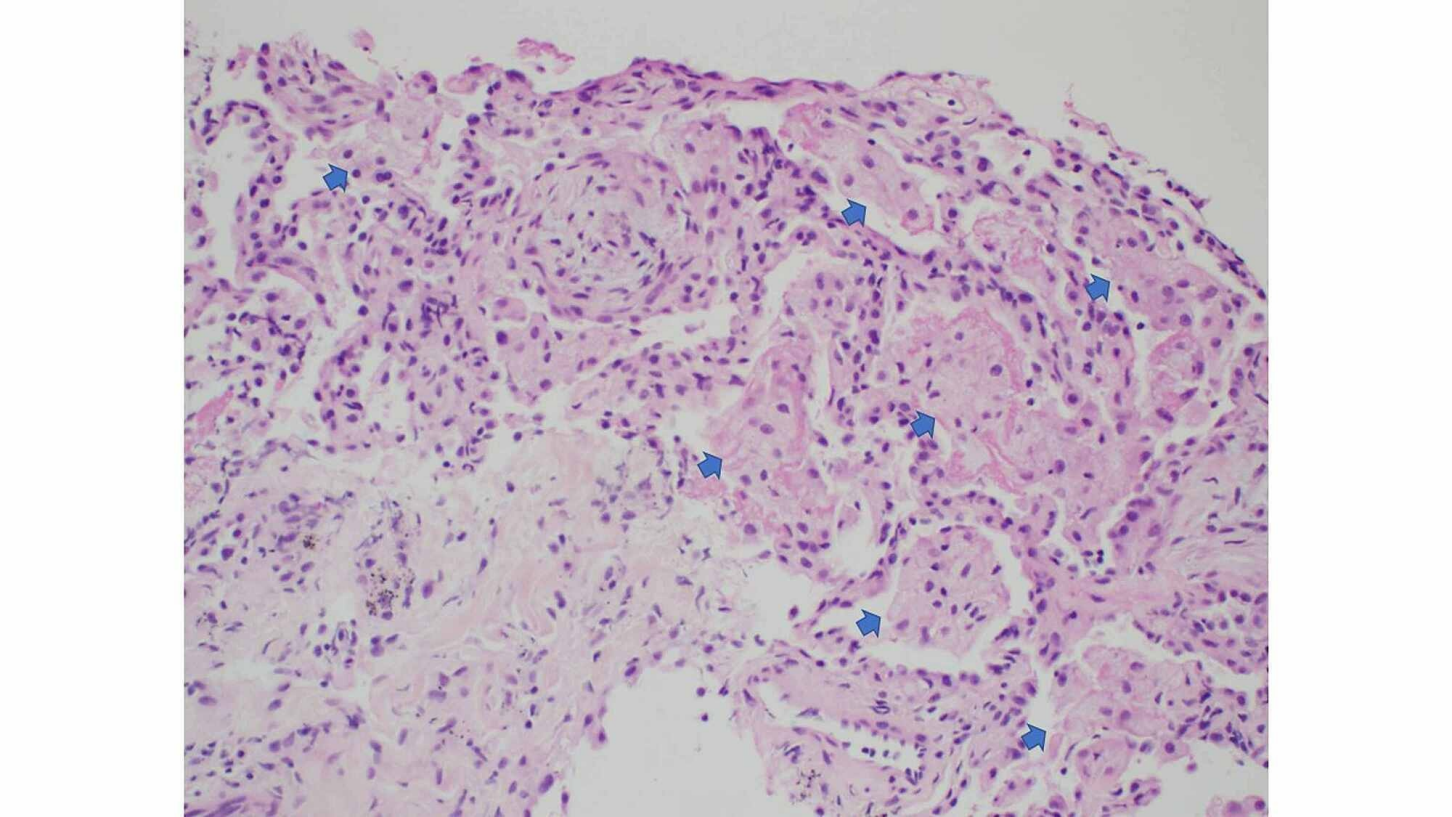
Lung biopsy showing foamy macrophages within alveolar space Arrows in the image point towards foamy macrophages which are present within alveolar spaces.

A 38-year-old male to female transgender woman presented to the emergency room with complaints of fever, nausea, vomiting, diarrhea, anorexia and generalized weakness for a week. The patient has a past medical history of HIV for 15 years and Classic Hodgkin’s Lymphoma that was diagnosed one year ago and completed six cycles of brentuximab vedotin plus doxorubicin, vinblastine, and dacarbazine (A+AVD) chemotherapy three months ago. Complete remission was confirmed by PET-CT scan after four cycles of chemotherapy. Initial vital signs showed blood pressure 92/40 mmHg, heart rate of 114 beats per minute, temperature 102.6 F, respiratory rate 22 bpm and oxygen saturation on room air 94%. Physical examination was positive for diffuse abdominal tenderness, male genitalia with multiple small round ulcerative lesions. Rest of the physical examination was unremarkable. Initial lab work showed white blood cells (WBC) 2930/mcL (normal range, 4800-1100/mcl), hemoglobin (Hb) 8.6 g/dL (normal range, 12-16 g/dL), platelets 21000/mcL (normal range, 150000-450000/mcL), sodium 125 mmol/L (normal range, 135-145 mmol/L), potassium 3.8 mmol/L (normal range, 3.5-5.1 mmol/L), calcium 6.7 mg/dL (normal range, 8.6-10.3 mg/dL), Albumin 2.4 g/dL (normal range, 3.5-5.2 g/dL), total bilirubin 2.5 mg/dL (normal range, 0.0-1.2 mg/dL), direct bilirubin 1.7 mg/dL (normal range, 0.0-03 mg/dL), alkaline phosphatase 153 U/L (normal range, 35-104 U/L), alanine transaminase (ALT) 64 U/L (normal range, 0-33 U/L) and aspartate transaminase (AST) 64 U/L (normal range, 5-32 U/L). CD4 count 96/mcL (normal range, 503-1144 cells/mcL) and CD8 count 663 cells/mcL (normal range, 279-578 cells/mcL). COVID-19 polymerase chain reaction (PCR) was negative. HIV viral load was undetectable. Urine and blood cultures came back negative. CT chest showed right middle lobe consolidation, right hilar and mediastinal lymphadenopathy (Figure 1). CT abdomen showed hepatosplenomegaly with hypodense lesion in liver and spleen and retroperitoneal lymphadenopathy (Figure 2). The patient was given broad spectrum antibiotics and adequately resuscitated with IV fluids but mean arterial pressure remained below 65 mmHg and the patient was started on vasopressors and systemic steroids for septic shock. Home medications for HIV Symtuza and dapsone were resumed. Patient’s symptoms improved initially and she became afebrile but pancytopenia persisted and the total and direct bilirubin level remained elevated. Genital ulcers swab PCR was positive for HSV-2 and the patient was treated with valacyclovir. Vasopressors and stress dose steroids were discontinued after two and six days respectively. On day 9 of hospital admission, the patient again started to spike high grade fevers despite being on antibiotics. Sputum culture came back positive for Mycobacterium avium complex which was treated with amikacin and azithromycin with no improvement in symptoms. Additional lab work showed EBV viral load 61 IU/mL (normal range ≤ 0 IU/L), high sensitivity C-reactive protein (CRP) 300 mg/L (normal range, <5 mg/L), activated partial thromboplastin time (APTT) 48.3 (normal range, 25.1-36.5 seconds), international normalised ratio (INR) 2, fibrinogen 488 mg/dL (normal range, 200-393 mg/dL), lactate dehydrogenase 198 U/L (normal range, 50-242 U/L), ferritin 10845 ng/mL (normal range, 15-150 ng/mL), interleukin-6 298.4 pg/mL (normal range, 0.0-15.5 pg/mL) and soluble interleukin-2 receptor 6831 pg/mL (normal range, 175.3-858.2 pg/mL), fasting triglyceride level 143 mg/dL (normal range, 10-149 mg/dL). Biopsy of the right lung opacity showed foamy macrophages in alveolar space and fibroblast proliferation suggestive of organizing pneumonia (Figure 3). Fluorescence in situ hybridization (FISH) analysis of bone marrow aspirate was negative for the lymphoma panel and chromosomal analysis showed normal male karyotype with 46 XY. Flow cytometry showed an increased percentage of natural killer-like T-cells, rest was unremarkable. The patient initially fulfilled five out of the eight criteria used in HLH-2004 study and was started on treatment with dexamethasone and etoposide under the presumptive diagnosis of HLH. Patient’s fever resolved and reported improvement in symptoms. Ferritin and soluble interleukin-2 receptors titrated down to 9052 ng/ml and 6042.2 ng/ml, respectively. Bone marrow biopsy later showed focal histiocytes with hemophagocytosis. Immunoperoxidase staining established the diagnosis of relapsed Classic Hodgkin’s Lymphoma. The patient was then transferred to a lymphoma program in a University hospital to start the treatment for underlying Hodgkin’s Lymphoma on a dedicated chemotherapy floor.

## Discussion

HLH is a serious medical condition associated with a high mortality rate. In our patient, multiple risk factors were present that can trigger HLH. The patient had a history of stage IV Classic Hodgkin’s Lymphoma that was treated by chemotherapy with A+AVD and achieved a PET-CT scan confirmed complete remission. HLH in the context of malignancy can occur in two settings. First and the more common is with concomitant active malignant disease whereas the second and less common when the patient has undergone or is currently undergoing chemotherapy [8]. The infectious trigger is usually identified in the second setting. Similarly EBV, HSV-2, HIV and MAC are all associated with HLH in literature [6,9,10]. Our patient fulfilled total six out of eight criteria as per HLH-2004 protocol [1], and received treatment with etoposide and dexamethasone for secondary HLH in the presence of risk factors such as prior chemotherapy, HIV, EBV, HSV-2 and MAC. Immunoperoxidase stain on bone marrow biopsy later established the diagnosis of relapsed Classic Hodgkin’s Lymphoma. It is important to recognize the underlying cause as the prognosis varies greatly in different underlying conditions. Many cases have been documented where multiple risk factors were present at the time of diagnosis of HLH and malignancy was diagnosed later on [5,11,12]. Gurunathan et al. reported a series of nine patients where diagnosis of HLH delayed the diagnosis of malignancy [12]. Most of the patients have infections at the time of diagnosis of HLH and HLH was wrongly attributed to infection. At the time of diagnosis of malignancy, seven out of nine patients were unable to receive chemotherapy due to severity of condition and multiple organ failure. The two patients that received chemotherapy were alive at the time of the conclusion of study. One retrospective analysis of adult patients concluded that average survival of malignancy-associated HLH was 1.2 months and non-malignancy associated HLH was 22.8 months [7]. Similarly another study in children and adolescents reported median survival of malignancy and chemotherapy-associated HLH 1.2 years and 0.9 years, respectively [8]. It should be kept in mind that many patients with HLH, like our patient, receive steroids under the impression of septic shock that also decrease the diagnostic yield of biopsy for lymphoproliferative disorder. In our patient, HIV was well controlled with undetectable viral load, EBV viral load was not very high, genital herpes was localized and chronic, and no clinical improvement with MAC treatment suggested that relapse of Classic Hodgkin’s Lymphoma was the underlying cause of HLH. As in our patient, the HLH with malignancy is usually treated by treating the HLH cytokine storm first and then treating the underlying malignancy as most of the time there is multiple organ injury at the time of diagnosis [13]. Mustafa Ali et al. reported a case of HLH in a patient with concurrent Hodgkin’s lymphoma, cytomegalovirus, EBV and Candida infection that was successfully treated by treating the cytokine storm first and Hodgkin’s lymphoma afterwards [5]. However, cases have been reported where malignancy-associated HLH is treated by treating the underlying malignancy [4,14]. Shaikh et al. reported a case of HLH associated with Hodgkin’s lymphoma, HIV and EBV that was successfully treated by treating the Hodgkin’s lymphoma directly and showed resolution of symptoms within a few days of chemotherapy [4]. This suggests that in patients presenting with HLH, extensive workup should be done to rule out active malignant conditions as malignancy-associated HLH is associated with poor prognosis and early diagnosis and treatment of the underlying malignant condition will improve the overall mortality and clinical outcomes.

## Conclusions

We present a case of HLH that we believe was triggered by relapse of recently treated Classic Hodgkin's Lymphoma. In such patients with a history of hematological malignancy, a high degree of suspicion for relapse should still be maintained even in the presence of other possible infectious triggers. We should also be cognizant of the fact that occasionally such patients would already be on empiric high dose steroids either for presumed septic shock or HLH which reduces the diagnostic yield of biopsy for lymphoproliferative disorders.

## References

[1] Henter JI, Horne A, Arico M (2007). HLH-2004: diagnostic and therapeutic guidelines for hemophagocytic lymphohistiocytosis. Pediatr Blood Cancer.

[2] Iardino A, Amar Z, Ahmed Y (2018). Epstein-Barr-positive classical Hodgkin lymphoma-associated haemophagocytic lymphohistocytiosis: a rare case. BMJ Case Rep.

[3] Booth AL, Osehobo P, Rodgers-Soriano D, Lalarukh A, Eltorky MA, Stevenson HL (2018). Hemophagocytic lymphohistiocytosis secondary to unknown underlying Hodgkin lymphoma presenting with a cholestatic pattern of liver injury. Case Rep Gastroenterol.

[4] Shaikh H, Shaikh S, Kamran A, Mewawalla P (2018). Cholestatic jaundice: a unique presentation leading to the diagnosis of HLH with Hodgkin lymphoma, HIV and EBV. BMJ Case Rep.

[5] Mustafa Ali M, Ruano Mendez AL, Carraway HE (2017). Hemophagocytic lymphohistiocytosis in a patient with Hodgkin lymphoma and concurrent EBV, CMV, and Candida infections. J Investig Med High Impact Case Rep.

[6] Lerolle N, Laanani M, Riviere S (2016). Diversity and combinations of infectious agents in 38 adults with an infection-triggered reactive haemophagocytic syndrome: a multicenter study. Clin Microbiol Infect.

[7] Parikh SA, Kapoor P, Letendre L, Kumar S, Wolanskyj AP (2014). Prognostic factors and outcomes of adults with hemophagocytic lymphohistiocytosis. Mayo Clin Proc.

[8] Lehmberg K, Sprekels B, Nichols KE (2015). Malignancy-associated haemophagocytic lymphohistiocytosis in children and adolescents. Br J Haematol.

[9] Yamaguchi K, Yamamoto A, Hisano M, Natori M, Murashima A (2005). Herpes simplex virus 2-associated hemophagocytic lymphohistiocytosis in a pregnant patient. Obstet Gynecol.

[10] Ordaya EE, Jarir SA, Yoo R, Chandrasekar PH (2017). Hemophagocytic lymphohistiocytosis (HLH): elusive diagnosis of disseminated Mycobacterium avium complex infection. Germs.

[11] Chan K, Behling E, Strayer DS, Kocher WS, Dessain SK (2008). Prolonged hemophagocytic lymphohistiocytosis syndrome as an initial presentation of Hodgkin lymphoma: a case report. J Med Case Rep.

[12] Gurunathan A, Boucher AA, Mark M (2018). Limitations of HLH-2004 criteria in distinguishing malignancy-associated hemophagocytic lymphohistiocytosis. Pediatr Blood Cancer.

[13] Daver N, McClain K, Allen CE (2017). A consensus review on malignancy-associated hemophagocytic lymphohistiocytosis in adults. Cancer.

[14] Hyun G, Robbins KJ, Wilgus N, Grosso L, Goyal SD (2016). Hemophagocytic lymphohistiocytosis in a patient with classical Hodgkin lymphoma. Case Rep Hematol.

